# Validity of the Trait Emotional Intelligence Questionnaire (TEIQue) in a Brazilian Sample

**DOI:** 10.3389/fpsyg.2022.735934

**Published:** 2022-03-24

**Authors:** Ana Carolina Zuanazzi, Gregory J. Meyer, Konstantinos V. Petrides, Fabiano Koich Miguel

**Affiliations:** ^1^Universidade São Francisco Programa de Pós-Graduação Stricto Sensu em Psicologia, São Paulo, Brazil; ^2^Department of Psychology, University of Toledo, Toledo, OH, United States; ^3^London Psychometric Laboratory, University College London, London, United Kingdom; ^4^Department of Psychology, Universidade Federal de São Carlos, São Paulo, Brazil

**Keywords:** emotional intelligence, psychometric properties, internal consistency, external validity, self-report measure

## Abstract

The study of the relationship between reasoning and emotional processes is not new in Psychology. There are currently two main approaches to understanding the aspects related to these processes called emotional intelligence: the ability model and the trait model. This study focuses on the latter, analyzing the factor structure, reliability, and validity of the Trait Emotional Intelligence Questionnaire (TEIQue) in a Brazilian sample. 4314 adults with ages ranging from 18 to 60 years (M = 30.37; SD = 9.73) answered the TEIQue and other online instruments measuring emotional regulation, impulsiveness, alexithymia, loneliness, quality of life, positive and negative affect, personality traits, emotional perception, emotional understanding, and reasoning tests. The original four-factor structure of the TEIQue was replicated, Cronbach’s alphas ranged from 0.60 to 0.89 for the facets, and from 0.76 to 0.90 for the factors and global score. The correlations followed theoretically expected directions, showing a stronger pattern for self-report measures than for performance tasks. Our results corroborated previous studies with the TEIQue, confirming the psychometric adequacy of the instrument in the Brazilian context. Future studies may focus on participants with lower levels of education and additional external criteria, such as career preferences, job performance, and health.

## Introduction

The study of the relationship between reasoning and emotional processes is not new in Psychology. In the early 20th century, Thorndike (1920) proposed the concept of social intelligence as the ability to correctly understand and interact with other people. Since then, multiple theories and models regarding cognition were developed to better understand this relationship, such as Gardner’s (1983) multiple intelligences model that included interpersonal (self) and intrapersonal (others) intelligence. Basically, these theories proposed the ability to understand one’s and other’s moods, intentions, and motivations and to behave properly in interpersonal relations (for a review, see Salovey and Mayer, 1990). Despite the importance of these theories, the lack of adequate instruments to operationalize them contributed to their abandonment.

In addition, a clearer and more objective conceptualization of a construct that integrated reasoning and emotions was required since the early ones were too broad. In the early 1990’s, Salovey and Mayer (1990) proposed a four-factor emotional intelligence model that defined the construct as the ability to perceive, monitor, and discriminate one’s own and others’ feelings and emotions, and to use this emotional information to guide one’s thinking and actions. The authors subsequently developed performance-based measures to assess these abilities (Mayer et al., 2016) which showed expected associations with reasoning tests as small-to-medium correlations and near-zero correlations with self-reported personality traits (e.g., Olderbak et al., 2018). As a cognitive ability, the construct is currently being studied as a factor in the 2nd stratum of intelligence in the Cattel-Horn-Carrol (CHC) theory perspective (e.g., Schneider and McGrew, 2018).

In parallel, other emotional intelligence models were proposed with different factor structures (e.g., Bar-On, 2005; Petrides, 2010). These models focus on one’s perception of their own behaviors or abilities rather than their knowledge about emotional intelligence strategies. Although many of those models are in continuous study, the trait emotional intelligence model (trait EI or trait emotional self-efficacy), developed by Petrides et al. (2016), is arguably the most widely researched (see Pérez-González et al., 2020). Trait EI comprises the affective aspects of personality and is defined as a constellation of emotional perceptions assessed through questionnaires and rating scales (Petrides et al., 2007).

Trait EI has been studied in multiple contexts, including educational, clinical, and organizational settings. In educational contexts, trait EI is positively associated with measures of creativity and negatively related to class absence or violations of school rules. Trait EI is a positive predictor of well-being and mental health and a negative predictor of psychopathology in adults and self-harm in adolescents. In general, it is negatively associated with stress, anxiety, and depression in adults. Finally, it is positively related to job performance, work-related well-being, and job satisfaction in the workplace. It is also negatively associated with job stress and burnout (for a review, see Petrides et al., 2016).

Despite criticism about trait EI having high associations with other personality traits, resulting in construct redundancy (Schulte et al., 2004), a meta-analysis demonstrated the incremental validity of this model measured by two self-report forms, the Trait Emotional Intelligence Questionnaire (TEIQue) and the Trait Emotional Intelligence Questionnaire-Short Form (TEIQue-SF; Andrei et al., 2016). Additionally, other studies showed that trait EI is a distinct and powerful explanatory construct (e.g., Van der Linden et al., 2017).

The TEIQue and TEIQue-SF are part of a set of measures based on the trait EI model that include questionnaires for children, adolescents, and adults (Petrides, 2009). The TEIQue has been adapted to other languages and studied regarding its validity, showing similar results to the original version. International studies include German-speaking Austria (Freudenthaler et al., 2008), Chinese-speaking Hong Kong, English-speaking Hong Kong, and English-speaking UK (Gökçen et al., 2014), Catalan (Aluja et al., 2016), Italian (Chirumbolo et al., 2019), Turkish (Ulutas, 2019), Lebanese (Sanchez-Ruiz et al., 2021), among others. To date, only the short form of the questionnaire is available in Brazil has been studied (Perazzo et al., 2021). The goal of the present research was to study the Brazilian- Portuguese TEIQue version in a large Brazilian sample, analyzing its factor structure, reliability, and validity by correlating it with other related measures. Considering previous findings, we expected the TEIQue to display moderate to high correlations with self-report measures of personality traits, especially those related to emotional experience, and low correlations with performance measures of emotional intelligence and intelligence.

## Materials and Methods

### Participants

Participants were 4,314 Brazilian individuals, with ages ranging from 18 to 60 years (*M* = 30.37; *SD* = 9.73), 54.6% women. Regarding education, 5.2% had attended up to middle school, 39.5% had attended up to high school, 41.9% had attended up to college, and 13.5% had attended graduate school.

### Measures

#### Trait Emotional Intelligence Questionnaire

The TEIQue comprises 153 items rated on a 7-point Likert scale ranging from 1 (“Strongly disagree”) to 7 (“Strongly agree”). It yields scores on 15 facets, 4 factors, and global trait EI. The Well-being factor includes the facets of Self-esteem (indicating self-confidence), Trait happiness (satisfaction with life), and Trait optimism (hopefulness). The Sociability factor includes the facets of Social awareness (social skills), Emotion management (influencing other people’s feelings), and Assertiveness (standing up for one’s rights). The Emotionality factor includes the facets of Emotion perception (including self and others), Emotion expression (communication of feelings), Relationships (interpersonal skills), and Trait empathy (taking other people’s perspective). Finally, the Self-control factor includes the facets of Emotion regulation (emotional control), Stress management (stress control), and Impulse control (low impulsiveness). In addition, two of the 15 facets are not included in a factor: Adaptability (flexibility and adaptation to new situations) and Self-motivation (will to persist).

#### Difficulties in Emotion Regulation Scales (DERS-16)

The DERS is a self-report inventory that assesses challenges in the ability to regulate one’s emotions, such as non-acceptance of emotional responses, difficulties engaging in goal-oriented behaviors, difficulties controlling impulses, lack of regulation strategies, and low emotional clarity. The scale was adapted and validated in Brazil (Miguel et al., 2017), with empirical evidence supporting both the 36- and 16-item versions. On our sample, Cronbach’s alpha for the 16-item DERS global score was 0.92.

#### Barratt Impulsiveness Scale

The BIS is a 30-item self-report inventory that assesses aspects of impulsiveness, such as non-inhibition of unsuited behavior, imprudent decision taking, and lack of planning. The scale was adapted and validated in Brazil (Malloy-Diniz et al., 2010). On our sample, Cronbach’s alpha for the BIS global score was 0.82.

#### Toronto Alexithymia Scale (TAS-20)

The TAS-20 is a 20-item self-report inventory that assesses alexithymia, i.e., difficulties in understanding one’s own feelings and using symbolic thinking. The scale was adapted and validated in Brazil (Colombarolli et al., 2019). On our sample, Cronbach’s alpha for the TAS global score was 0.84.

#### The Revised UCLA Loneliness Scale

The UCLALS-BR is a 20-item self-report inventory that assesses the experience of negative emotions due to loneliness and social isolation. The scale was adapted and validated in Brazil (Barroso et al., 2016). On our sample, Cronbach’s alpha for the UCLALS-BR global score was 0.94.

#### World Health Organization Quality of Life

The WHOQOL is a 26-item self-report inventory developed by the World Health Organization to assess quality of life in several contexts. The brief version was adapted and validated in Brazil (Fleck et al., 2000). For the purposes of the present research, the Psychological and Social life quality scales were used. On our sample, Cronbach’s alpha for the two scales were 0.82 (Psychological) and 0.70 (Social).

#### Positive and Negative Affect Schedule

The PANAS is an inventory that lists 10 positive and 10 negative emotional states. Participants rate how frequently they felt each emotion recently on a 5-point Likert scale. The scale was adapted and validated in Brazil (Zanon et al., 2013). On our sample, Cronbach’s alpha for positive affect was 0.76, and 0.85 for negative affect.

#### Clinical Dimensional Personality Inventory Version 2

The IDCP-2 is a self-report inventory that assesses pathological personality traits according to Millon’s personality theory and DSM-IV-TR’s axis II (Carvalho and Primi, 2015). For the present research, only two factors that are specific to emotional experience were used: Mood Instability (frequent changes in mood), and Criticism Avoidance (withdrawal from social contact because of fear of negative emotions). On our sample, Cronbach’s alpha was 0.88 for Mood Instability and 0.89 for Criticism Avoidance.

#### Computerized Test of Primary Emotions Perception

The PEP is a performance measure of emotional perception that displays 38 brief videos of people expressing emotions (with the first three videos used as examples of the task). Participants watch each video and choose which emotions they believe are present, from a list of eight: joy, love, fear, surprise, sadness, disgust, anger, and curiosity. The PEP was developed in Brazil and showed adequate validity (Miguel and Primi, 2014). The test is scored using the Rasch model, and the reliability on our sample was 0.61.

#### Emotional Understanding (CE, Conhecimento Emocional, in Portuguese)

The CE is a 30-item performance measure of the ability to recognize how emotions arise, blend and change over time. It displays short stories of characters in different situations, and participants must choose the alternative that correctly displays the sequence of experienced emotions (Peixoto et al., 2019). On our sample, Cronbach’s alpha for this measure was 0.68.

#### Battery of Reasoning Tests (BPR-5)

The BPR-5 is a set of five intelligence tasks. For the present research, we used two tests: abstract reasoning (AR), which assesses fluid intelligence, and verbal reasoning (VR), which assesses both fluid intelligence and vocabulary (Primi and Almeida, 2000). On our sample, Cronbach’s alpha for AR was 0.78 and for VR it was 0.68.

### Procedures

The English version of the TEIQue was translated by two researchers with previous experience in test development and adaptation. The researchers reviewed their translations, resolving discrepancies, until a final Brazilian-Portuguese version of the TEIQue was obtained.

All tests were adapted for online use (except for PEP, which was originally designed to be administered online) and uploaded to a specific domain for the research. The research was approved by the State University of Londrina’s Ethical Committee (approval number 64469717.6.0000.5231), and participation in research followed the committee, the International Test Commission and Helsinki Declaration guidelines. Participants were invited from a popular social media platform (Facebook) and were shown the Consent Form. If they agreed to participate, they created a unique user account, to avoid duplicate responses. Due to the high number of instruments, administrations followed an alternating method. The TEIQue was always presented first; the order of the other assessments was changed periodically, while still being able to answer all tests, if they wanted. Because of this, not all participants completed all assessments. In fact, only 9% of participants answered more than two assessments in addition to the TEIQue. This design was preferred in order to reduce fatigue effects. The number of participants that answered each assessment individually is displayed in Table 4. In accordance with the Ethical Committee’s guidelines, no incentives were offered to the participants, and all had the option of opting out of participation.

### Data Analyses

Exploratory factor analysis was conducted using the TEIQue facets in a principal component factoring with Oblimin rotation. We retained factors based on parallel analysis, considering 15 variables, 4,314 participants, and 100 randomly generated correlation matrices. Despite the fact that this factor structure has already been established in other studies, we chose exploratory over confirmatory factor analysis for the sake of consistency and replication since the former approach has been used in most international studies with the TEIQue. In addition, exploratory factor analysis allowed us to verify any configuration different than the other international studies.

Reliability for facets, factors, and the total score were estimated through Cronbach’s alpha. To investigate the relationship of the TEIQue with other measures, Pearson correlations were calculated. Correlation coefficients were interpreted as low around 0.10, moderate around 0.30, and strong around 0.50 (Cohen, 1992).

## Results

The exploratory factor analysis showed a KMO index of 0.89, indicating data adequacy. Table 1 shows the eigenvalues from the factor analysis along with those from the parallel analysis (only the first five components are displayed).

**TABLE 1:** Parallel analysis results.

Component	Eigenvalue	Cumulative variance explained	Parallel analysis
1	6.43	42.87%	1.10
2	1.69	54.13%	1.08
3	1.17	61.96%	1.06
4	1.06	68.99%	1.05
5	0.84	74.58%	1.04

Four factors were obtained, replicating the original four-factor structure. In addition, Self-motivation and Adaptability, two facets that traditionally are not included in the factors, presented considerable loadings on Well-being and Self-control, respectively. Factor loadings are presented in Table 2.

**TABLE 2:** Factor pattern matrix for the TEIQue facets.

	Well-being	Sociability	Emotionality	Self-control
Happiness	**0.90**	–0.11	–0.06	0.03
Optimism	**0.85**	–0.07	–0.06	0.07
Self-motivation	0.68	0.13	–0.01	0.11
Self-esteem	**0.64**	0.31	0.06	0.14
Emotion management	–0.20	**0.85**	–0.15	0.12
Assertiveness	0.26	**0.78**	0.21	–0.04
Social awareness	0.18	**0.64**	–0.22	0.15
Empathy	–0.09	–0.05	−**0.84**	0.13
Emotion expression	0.26	0.28	−**0.60**	–0.24
Relationships	0.33	–0.18	−**0.55**	0.23
Emotion perception	0.13	0.36	−**0.52**	0.06
Emotion regulation	–0.01	0.15	0.13	**0.88**
Stress management	0.04	–0.07	–0.05	**0.85**
Impulse control	0.08	–0.06	–0.03	**0.68**
Adaptability	0.06	0.10	–0.14	0.52

*Keyed factor loadings (see Petrides, 2009) are presented in bold.*

Descriptive statistics for the TEIQue scores are displayed in Table 3, including reliability information, and means and standard deviations broken down by sex. Cronbach’s alphas for the facets ranged from 0.60 to 0.89, while alphas for the factors and global score ranged from 0.76 to 0.90. The means of facets and factors tended to be around 4.50 with standard deviation around 1.00. Sex differences were mostly small (with Cohen’s *ds* below 0.19), except for the facets of Emotion regulation and Stress management, and the factor of Self-control, where men scored moderately higher (*d* = 0.49, 0.49, 0.43, respectively).

**TABLE 3:** Descriptive statistics (means and standard deviations) for the TEIQue variables.

	Cronbach’s alpha	Global sample	Skewness (SE)	Kurtosis (SE)	Women’s mean and SD (*n* = 2353)	Men’s mean and SD (*n* = 1890)
**Facets**						
Adaptability	0.65	4.18 (0.90)	−0.14 (0.04)	−0.04 (0.07)	4.12 (0.92)	4.25 (0.89)
Assertiveness	0.64	4.53 (0.94)	−0.13 (0.04)	−0.09 (0.07)	4.48 (0.97)	4.60 (0.92)
Emotional expression	0.85	4.11 (1.36)	0.06 (0.04)	−0.67 (0.07)	4.20 (1.38)	3.98 (1.34)
Emotional management	0.75	4.62 (1.07)	−0.14 (0.04)	−0.38 (0.07)	4.53 (1.07)	4.73 (1.07)
Emotional perception	0.77	4.55 (1.08)	−0.3 (0.04)	−0.39 (0.07)	4.57 (1.09)	4.53 (1.08)
Emotional regulation	0.80	4.17 (1.03)	−0.07 (0.04)	−0.18 (0.07)	3.95 (1.01)	4.45 (1.00)
Impulse control	0.76	4.18 (1.14)	−0.05 (0.04)	−0.43 (0.07)	4.13 (1.15)	4.25 (1.11)
Relationships	0.60	4.81 (0.96)	−0.32 (0.04)	−0.16 (0.07)	4.85 (0.98)	4.76 (0.92)
Self-esteem	0.80	4.60 (1.07)	−0.47 (0.04)	−0.3 (0.07)	4.54 (1.07)	4.68 (1.06)
Self-motivation	0.76	4.62 (1.04)	−0.22 (0.04)	−0.31 (0.07)	4.65 (1.06)	4.59 (1.02)
Social awareness	0.82	4.59 (1.10)	−0.19 (0.04)	−0.27 (0.07)	4.54 (1.09)	4.66 (1.10)
Stress management	0.83	4.04 (1.21)	−0.10 (0.04)	−0.59 (0.07)	3.79 (1.21)	4.36 (1.14)
Trait empathy	0.74	4.97 (1.02)	−0.53 (0.04)	0.38 (0.07)	5.05 (0.99)	4.87 (1.05)
Trait happiness	0.89	4.92 (1.42)	−0.56 (0.04)	−0.39 (0.07)	4.95 (1.43)	4.88 (1.40)
Trait optimism	0.83	4.82 (1.24)	−0.46 (0.04)	−0.34 (0.07)	4.86 (1.26)	4.78 (1.23)
**Factors**						
Well-being	0.86	4.78 (1.10)	−0.54 (0.04)	−0.22 (0.07)	4.78 (1.12)	4.78 (1.09)
Self-control	0.79	4.13 (0.95)	−0.03 (0.04)	−0.26 (0.07)	3.96 (0.95)	4.35 (0.91)
Emotionality	0.76	4.61 (0.85)	−0.04 (0.04)	−0.17 (0.07)	4.67 (0.85)	4.54 (0.85)
Sociability	0.80	4.58 (0.88)	−0.13 (0.04)	−0.14 (0.07)	4.52 (0.88)	4.66 (0.88)
Global trait EI	0.90	4.51 (0.72)	−0.08 (0.04)	−0.17 (0.07)	4.48 (0.74)	4.56 (0.70)

**TABLE 4:** Correlations between key variables in the study.

	Well-being	Sociability	Emotionality	Self-control	Global trait EI
**TEIQue (*N* = 4,314)**					
Well-being		0.50**	0.59**	0.52**	0.85**
Sociability			0.53**	0.32**	0.72**
Emotionality				0.43**	0.82**
Self-control					0.72**
DERS-16 (*n* = 311)	−0.58**	−0.38**	−0.42**	−0.64**	−0.65**
BIS (*n* = 283)	−0.41**	−0.21**	−0.32**	−0.65**	−0.51**
TAS-20 (*n* = 252)	−0.52**	−0.44**	−0.62**	−0.38**	−0.64**
UCLALS-BR (*n* = 283)	−0.59**	−0.38**	−0.41**	−0.35**	−0.57**
**WHOQOL (*n* = 294)**					
Psychological	0.79**	0.39**	0.43**	0.53**	0.70**
Social	0.53**	0.28**	0.42**	0.27**	0.49**
**PANAS (*n* = 250)**					
Positive	0.55**	0.46**	0.37**	0.23**	0.54**
Negative	−0.54**	−0.34**	−0.33**	−0.41**	−0.53**
**IDCP-2 (*n* = 243)**					
Mood instability	−0.73**	−0.21*	−0.27**	−0.67**	−0.65**
Criticism avoidance	−0.58**	−0.36**	−0.47**	−0.37**	−0.62**
PEP (*n* = 386)	–0.04	0.03	0.00	–0.02	–0.02
CE (*n* = 316)	0.01	0.05	0.19*	0.06	0.10
AR (*n* = 389)	–0.01	–0.01	0.05	0.21**	0.07
VR (*n* = 437)	0.05	0.11*	0.08	0.13*	0.11*

*p < 0.01; **p < 0.01.

The TEIQue’s four factors and global score were correlated with the other instruments. In addition, as theoretically expected (Petrides, 2009), the TEIQue factor scores were strongly intercorrelated. Results are presented in Table 4. Correlations with self-report measures were all significant, displaying moderate-to-strong effect sizes. In contrast, correlations with the performance tasks tended to be low and mostly non-significant, although the comparatively lower reliabilities for the latter set of measures ought to be taken into account in the evaluation of these results.

## Discussion

The goal of this research was to investigate the psychometric properties and validity of the Brazilian adaptation of TEIQue. The factor structure and reliability indices we found were similar to those reported in other international studies (Freudenthaler et al., 2008; Gökçen et al., 2014; Aluja et al., 2016). Alpha indices tended to be lower at the facet than the factor level, suggesting a more robust assessment using the TEIQue factors of the full form. In addition, the Brazilian means for the facet, factor and global score tended to be similar to those from other countries, indicating a similar distribution of the trait level across Western cultures. Similar results for the short form of Brazilian Portuguese TEIQue was found for the factor and global score (Perazzo et al., 2021). In the same line from those international studies, we found gender differences for some factors, suggesting distributions of emotional regulation, stress management and self-control that are different between men and women.

We studied TEIQue’s factor structure using exploratory factory analysis, which showed a structure that is similar to that found in other international studies. While a confirmatory factory analysis could have been conducted, given that the factor structure was previously established, we decided to use the exploratory method for the sake of replicability. Furthermore, although the analysis supported the general structure, a surprising result was also found, which perhaps could not have been found with confirmatory analysis. The facets of Self-motivation and Adaptability loaded on the factors of Well-being and Self-control, respectively. Similar results were reported by Aluja et al. (2016); Catalan adaptation and by Freudenthaler et al. (2008); German adaptation. However, in their samples, those facets also showed moderate loadings on other factors, while in our sample the cross-loadings were low. In fact, Self-motivation even showed a slightly higher loading on the Well-being factor than Self-esteem, suggesting that persistence and determination could also be considered specific aspects of Well-being. Likewise, flexibility to new situations could be considered a specific aspect of emotional control. This result is corroborated by other studies that have shown dispositional optimism and motivation are related to well-being (Hanssen et al., 2015), and that the ability to select from several coping strategies (flexibility) is an important aspect of emotional regulation (Aldao et al., 2015). Nevertheless, following the standard TEIQue scoring procedures, we did not include these two facets in the calculation of the factors, although future studies (which may include confirmatory analysis of different structures) may consider including them, as our results suggest a possibly – albeit slightly – different factor configuration in the Brazilian population.

All TEIQue factors showed strong intercorrelations, with the exception of the correlation between Sociability and Self-control, which was moderate. Still, taking these results together with the factor and reliability analyses, it is evident that the TEIQue displayed adequate internal consistency, replicating the same factor structure found in previous studies.

Our results also corroborated previous studies that showed trait emotional intelligence as measured by the TEIQue correlates mildly or strongly with other measures of personality traits (Petrides et al., 2016; Van der Linden et al., 2017). While all TEIQue correlations with personality variables were statistically significant, they did vary in magnitude. As expected, measures of emotional dysregulation and impulsiveness showed strong negative correlations with the TEIQue Self-control factor, which concerns control of impulses and stress. Alexithymia also correlated strongly with the Emotionality factor, which concerns perception and expression of emotions. Loneliness, quality of life, and frequency of positive and negative affects correlated strongly with TEIQue Well-being factor, which concerns happiness and general positivity.

Finally, our results were also in agreement with research showing that trait emotional intelligence correlates to a low degree with performance measures of emotion and reasoning. The only exception was in the correlation between abstract reasoning and the TEIQue Self-control factor (*r* = 0.21), which broadly echoed findings of low-to-moderate, yet statistically significant, associations between fluid intelligence and neuroticism (emotional stability), reported in other studies (e.g., Di Fabio and Palazzeschi, 2015).

A few limitations of this study should be considered. Although our sample was representative of all 26 Brazilian states and federal districts, the participants’ level of schooling was considerably higher than average. Further studies should verify the validity of the TEIQue in samples with lower levels of education, considering that nearly half of Brazilian population have not completed high school.

In addition, our study was conducted purely online, with no use of paper-and-pencil instruments. Although most studies show that online and printed versions of inventories tend to show almost identical psychometric parameters (Weigold et al., 2013), a formal equivalence study is advised. Finally, we also recommend further studies with other external criteria from the domains of career choice, work, and health, among others. The results of our study strongly suggest that this further recommended research can be confidently conducted with the current Brazilian adaptation of the full form of the Trait Emotional Intelligence Questionnaire (TEIQue).

## Data Availability Statement

The datasets presented in this article are not readily available because the regulations on research with humans by the Brazilian ethics councils prevent collected data from being made public without a specific request. Requests to access the datasets should be directed to AZ, anacarolina.zf@gmail.com.

## Ethics Statement

The studies involving human participants were reviewed and approved by participation in research followed guidelines from the State University of Londrina’s Ethical Committee and the International Test Commission and followed Helsinki Declaration guidelines. The patients/participants provided their written informed consent to participate in this study.

## Author Contributions

All authors listed have made a substantial, direct, and intellectual contribution to the work, and approved it for publication.

## Conflict of Interest

The authors declare that the research was conducted in the absence of any commercial or financial relationships that could be construed as a potential conflict of interest.

## Publisher’s Note

All claims expressed in this article are solely those of the authors and do not necessarily represent those of their affiliated organizations, or those of the publisher, the editors and the reviewers. Any product that may be evaluated in this article, or claim that may be made by its manufacturer, is not guaranteed or endorsed by the publisher.
